# Modulating effects of crocin on lipids and lipoproteins: Mechanisms and potential benefits

**DOI:** 10.1016/j.heliyon.2024.e28837

**Published:** 2024-04-03

**Authors:** Habib Yaribeygi, Mina Maleki, Farin Rashid-Farrokhi, Payman Raise Abdullahi, Mohammad Amin Hemmati, Tannaz Jamialahmadi, Amirhossein Sahebkar

**Affiliations:** a Research Center of Physiology, Semnan University of Medical Sciences, Semnan, Iran; b Urology and Nephrology Research Center, Shahid Beheshti University of Medical Sciences, Tehran, Iran; c CKD Research Centre, Shahid Beheshti University of Medical Science, IranNephrology Department, Masih Daneshvari Hospital, Telemedicine Research Center, National Research Institute of Tuberculosis and Lung Disease, Tehran, Iran; d Student Research Committee, Semnan University of Medical Sciences, Semnan, Iran; e Pharmaceutical Research Center, Pharmaceutical Technology Institute, Mashhad University of Medical Sciences, Mashhad, Iran; f Medical Toxicology Research Center, Mashhad University of Medical Sciences, Mashhad, Iran; g Biotechnology Research Center, Pharmaceutical Technology Institute, Mashhad University of Medical Sciences, Mashhad, Iran; h Applied Biomedical Research Center, Mashhad University of Medical Sciences, Mashhad, Iran

**Keywords:** Diabetes mellitus, Lipid, Crocin, Cholesterol, Adipogenesis, Lipolysis, Lipid peroxidation

## Abstract

Dyslipidemia poses a significant risk to cardiovascular health in both diabetic and non-diabetic individuals. Therefore, it is crucial to normalize lipid homeostasis in order to prevent or minimize complications associated with dyslipidemia. However, pharmacological interventions for controlling lipid metabolism often come with adverse effects. As an alternative, utilizing herbal-based agents, which typically have fewer side effects, holds promise. Crocin, a naturally occurring nutraceutical, has been shown to impact various intracellular pathways, reduce oxidative stress, and alleviate inflammatory processes. Recent evidence suggests that crocin may also confer lipid-related benefits and potentially contribute to the normalization of lipid homeostasis. However, the specific advantages and the cellular pathways involved are not yet well understood. In this review, we present the latest findings regarding the lipid benefits of crocin, which could be instrumental in preventing or reducing disorders associated with dyslipidemia. Additionally, we explore the potential cellular mechanisms and pathways that mediate these lipid benefits.

## 1 Introduction

The prevalence of diabetes mellitus (DM) is rapidly increasing globally [1], and it has detrimental effects on various physiological systems, leading to diabetic complications [2]. DM is a chronic metabolic disorder that negatively impacts the metabolism of multiple substrates, including lipids, resulting in dyslipidemia [3,4]. Dyslipidemia is a significant risk factor for complications such as cardiovascular disorders and renal failure, underscoring the importance of normalizing lipid metabolism in the diabetic context [[4], [5], [6]]. While several pharmacological agents have been developed to regulate lipid metabolism, they often come with adverse effects [7,8]. Consequently, researchers have explored the use of herbal-based nutraceuticals to provide lipid benefits in the diabetic setting [[9], [10], [11]]. Crocin, a naturally occurring pigment primarily found in saffron (*Crocus sativus*), has shown pharmacological effects in the diabetic milieu [[12], [13], [14]]. There is also evidence suggesting that crocin can modulate and normalize lipid metabolism, thereby improving dyslipidemia in the diabetic context [[15], [16], [17]]. There are numerous conventional and newer lipid-lowering therapies currently available in the market [[18], [19], [20], [21]]. The mainstay of treating dyslipidemia are statins, which possess not only cholesterol-lowering effects but also numerous pleiotropic properties [[22], [23], [24], [25], [26], [27], [28], [29], [30], [31], [32], [33]]. However, not all patients can tolerate statins [34,35] and this opens the avenues for research and development of new therapies for the considerable number of statin-intolerant patients. Plant-derived phytochemicals have been an attractive option for the discovery of lipid-modifying compounds owing to their potential safety, availability, and lower costs compared to biological agents [[36], [37], [38], [39], [40], [41], [42], [43], [44]]. Given the high prevalence of dyslipidemia and the considerable number of patients who are intolerant of statin therapy or resistant to current pharmacological agents, drug screening programs and pharmacological testing of bioactive phytochemicals are particularly important to introduce efficacious therapeutic options. Despite the promising potential of crocin, up-to-date evidence on the beneficial effects of this compound on lipid metabolism remains scarce. Therefore, this review aims to present the latest findings regarding the potential effects of crocin on lipid metabolism from a mechanistic perspective.

### 1.1 Saffron (*Crocus sativus*) and crocin

Saffron, a plant belonging to the Iridaceae family, has traditionally been used as a food additive and aromatic spice [45]. Moreover, historical documents highlight its potent therapeutic properties in various organs, as recognized by scientists such as Avicenna [46,47]. Saffron (*Crocus sativus*) is now cultivated in different countries worldwide, with approximately 90% of global production taking place in Iran [48]. Extracts from saffron and its active ingredients can modulate transcription factors, growth factors, and various intracellular signaling pathways [13,49,50]. Crocin, picrocrocin, and safranal are the main active beta-carotenoids isolated from saffron, responsible for its color, taste, and odor, respectively [51].

**Fig. 1:**
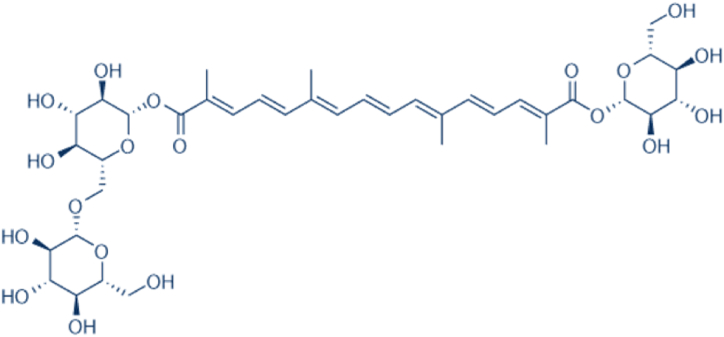
Chemical structure of crocin (C_44_H_64_O_24_).

Crocin, an expensive constituent of both saffron (*Crocus sativus*) and Gardenia plants, exhibits beneficial metabolic and extra-metabolic effects [[52], [53], [54], [55], [56]]. It has a molecular mass of 976.96 g/mol and a chemical formula of C_44_H_64_O_24_ (Fig. 1) [57]. Crocin belongs to a group of hydrophilic carotenoids that include monoglycosyl or diglycosyl polyene esters of crocetin, synthesized through the esterification of the disaccharide gentiobiose (composed of two d-glucose molecules joined by a β linkage) and crocetin [57]. This water-soluble beta-carotene possesses free radical scavenging properties due to its sugar moiety, making it a potent herbal-based antioxidant [52,58]. Additionally, crocin exhibits potent hypoglycemic effects, improving glycemic profiles and preventing hyperglycemia-induced pathophysiological pathways such as oxidative stress and fibrosis [13,59,60]. Evidence suggests that crocin exerts various pharmacological effects, including antioxidant properties [12], anti-inflammatory effects [13], memory enhancement [61,62], gene protection [63], anti-cough activity [64], anti-tumor effects [65], antidepressant properties [66], cardiovascular protection [67], neuroprotection [68], and potentiation of sexual behaviors [69,70]. Furthermore, recent studies suggest that crocin can modulate lipid metabolism, improve lipid profiles, and potentially prevent dyslipidemia-dependent vascular complications [60,71,72] (Table 1).

**Table 1:** Different impacts of crocin on lipid metabolism.

Pathways	Impacts of Crocin	Ref.
Lipolysis and Lipogenesis	Induce lipolysis and reduces adipogenesis via several pathways	[73,74,75,76]
Lipid Peroxidation	Potentiates anti-oxidant defense system and reduce lipotoxicity	[77,78,79]
Lipid Absorption/Transport and Cholesterol Metabolism	Reduce lipid absorption and circulating lipid and cholesterol levels	[80,76,81,82]

### 1.2 Lipid homeostasis in health and diseases

Lipids are hydrocarbon biomolecules that are insoluble in water but soluble in nonpolar solvents [83]. Due to differences in their structure, lipids exist in several forms, including triglycerides (TG), phospholipids (PL), cholesterol (CLS), and a few others of minor importance (such as sphingolipids, glycolipids, and prostaglandins) [84]. They play various physiological roles in the human body, such as energy storage, signaling activities, and structural functions [84]. Lipids are integral components of eukaryotic cell membranes, forming a double-layered lipid bilayer that encloses cells [84]. Additionally, they contribute to the structure of steroids such as vitamin D3, prostaglandins, sex hormones, and adrenal steroids (such as glucocorticoids and mineralocorticoids) [84]. Fats are synthesized, stored, metabolized, and consumed in the body on a daily basis [85]. They are distributed throughout the body but are primarily stored in adipose tissue (AT) [85]. Adipose tissue is a mass of fats, mainly consisting of triglycerides and phospholipids [83,86]. While it was previously considered an inert tissue responsible for storing excess energy, subsequent research has demonstrated that adipose tissue has additional biological activities, including the synthesis and release of important biomolecules called adipokines and adiponectins into the circulation [86]. These peptides have significant hormonal effects on metabolic pathways, leading to the recognition of adipose tissue as an endocrine organ [87].

Lipid metabolism encompasses the processes of absorption, synthesis, polymerization, conversion, and degradation of lipid molecules [83]. It is a tightly regulated procedure with a delicate dynamic equilibrium in the physiological state, where some lipids are being oxidized while others are being synthesized, replaced, and stored [83,88]. Lipid metabolism is influenced by various endogenous and exogenous factors and is regulated by growth hormone, sex steroids, adipokines, adrenal steroids, thyroid hormones, and neuronal stimuli [88,89]. Moreover, numerous physiological, pathological, and social factors, such as exercise, physical activity, feeding habits, and stressors, can modify lipid metabolism [89].

Lipid homeostasis plays emerging roles in overall body health and is involved in the normal functioning of various physiological systems, including the cardiovascular system, kidneys, retina, and nervous system [90]. On the other hand, dyslipidemia is closely associated with life-threatening disorders such as cancer, atherosclerosis, nephropathy, liver insufficiency, thrombosis, and heart attacks [90,91]. For example, hypercholesterolemia and hypertriglyceridemia are major underlying causes of atheroma plaque formation, atherosclerosis, and myocardial and cerebral infarctions [92]. Dyslipidemia also has negative impacts on renal function [93] and retinal function [94,95]. In the diabetic context, the conditions are particularly conducive to dyslipidemia and improper fat metabolism, as most diabetic complications are associated with dyslipidemia [91,96,97]. Consequently, many diabetic patients take lipid-controlling drugs, in addition to anti-diabetic medications, to improve their lipid profiles and prevent dyslipidemia-induced complications [98].

### 1.3 Crocin and Lipid homeostasis

Crocin is a potent nutraceutical with multiple effects on metabolism [99]. However, its precise effects on different pathways of lipid metabolism, such as lipogenesis, lipid peroxidation, and biosynthesis, are not well understood. Therefore, in the following sections, we will present the current knowledge about the potential benefits of crocin on lipid metabolism from a mechanistic perspective.

## 2 Lipogenesis and lipolysis

**Fig. 2:**
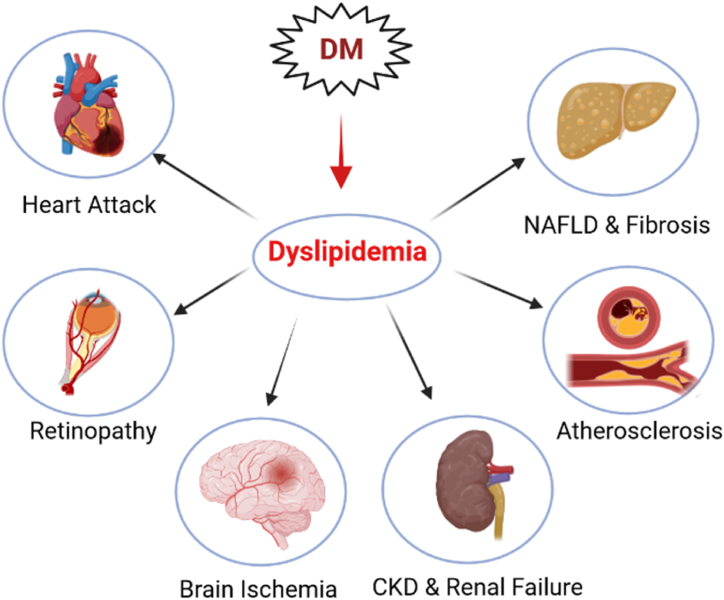
Diabetes-induced dyslipidemia is a major cause of many complications in other tissues (CKD = chronic kidney disease, NAFLD = non-alcoholic fatty liver disorder).

Lipogenesis and lipolysis are the two main processes involved in lipid homeostasis, with a highly controlled equilibrium that determines the total body fat mass [100]. Generally, during lipogenesis (or adipogenesis), free fatty acids (FFA) and triglycerides (TG) are produced from carbohydrates or acetyl-coenzyme A (CoA) in mitochondria and smooth endoplasmic reticulum (ER). On the other hand, during lipolysis, TGs break down into FFAs and glycerol through hydrolysis at the surface of cytosolic lipid droplets in adipocytes [100,101]. There exists a delicate balance between these two metabolic processes, but in pathological states, this balance is disrupted, leading to the development of dyslipidemia [101]. Excessive lipid synthesis or impaired lipolysis is associated with dyslipidemia and subsequent metabolic disorders, such as obesity, insulin resistance, diabetes mellitus (DM), non-alcoholic fatty liver disease (NAFLD), and atherosclerosis (Fig. 2) [[101], [102], [103]].

**Fig. 3:**
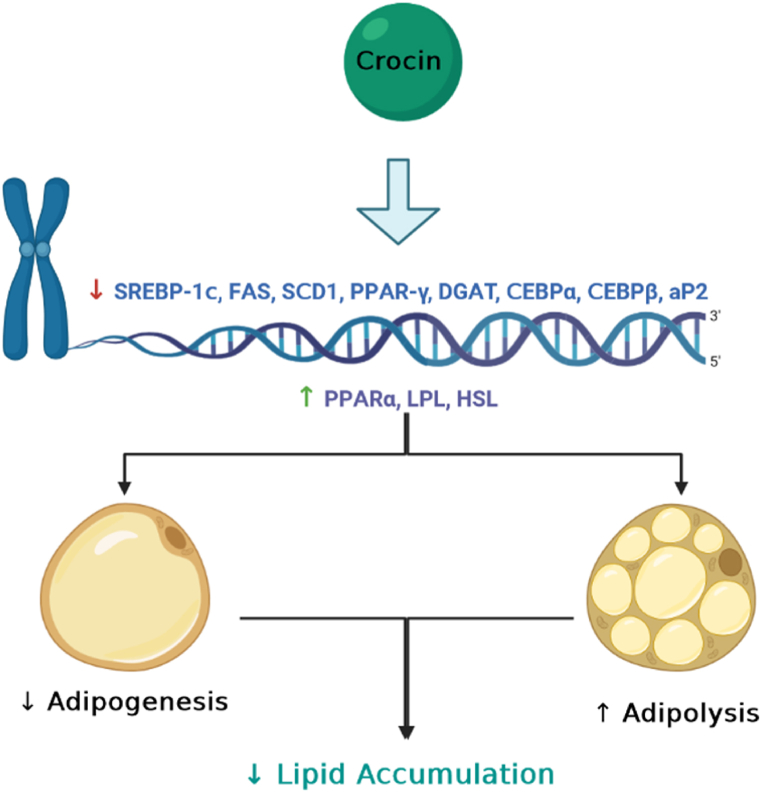
Crocin reduces gene expression of SREBP-1c (sterol regulatory element binding protein-1c), FAS (fatty acid synthase), SCD1 (stearoyl-CoA desaturase 1), PPAR-γ (peroxisome proliferator activated receptor-γ), DGAT (diacylglycerol acyltransferase), CEBPα (CCAAT/enhancer binding protein α), CEBPβ, aP2 (adipogenesis adiponectin) and up-regulates PPARα, LPL (lipoprotein lipase), and HSL (hormone-sensitive lipase) that in turn ameliorate adipogenesis and intensify adipolysis processes and attenuate lipid accumulation.

There is evidence suggesting that crocin decreases adipogenesis, increases lipolysis, and prevents lipid accumulation, making it a potential agent for reducing obesity in clinical settings (Fig. 3) [73,74]. Luo et al., in 2019 reported that oral administration of crocin for 8 weeks in db/db mice reduced the expression of genes involved in lipogenesis, including sterol regulatory element-binding protein-1c (SREBP-1c), fatty acid synthase (FAS), stearoyl-CoA desaturase 1 (SCD1), peroxisome proliferator-activated receptor-gamma (PPAR-gamma), and diacylglycerol acyltransferase (DGAT) [74]. Additionally, Xie et al., in 2022 administered crocin via oral gavage for 10 weeks to high-fat diet (HFD)-induced obese mice and observed a reduction in obesity [104]. They found that crocin decreases lipid synthesis in the liver under conditions of high-fat nutrition, improves inflammation and levels of short-chain fatty acids, and ameliorates lipid metabolism, resulting in decreased serum lipid levels [104]. In another study, oral administration of crocin for 12 weeks in mice fed a HFD led to a decrease in weight gain and lipid deposition, as well as an improvement in lipid profiles [80]. Lai and colleagues also demonstrated that 8 weeks of quercetin and crocin gavage reduced fat accumulation in the liver and lowered serum lipid levels in HFD and streptozotocin-induced type 2 diabetic rats [105].

We have limited evidence demonstrating that crocin can modulate the β-oxidation of FFAs [75]. β-oxidation of FFAs is a primary form of lipolysis [106]. It is a polyphasic catabolic process that occurs in mitochondria and peroxisomes, where FFAs are broken down to produce acetyl-CoA. This acetyl-CoA is then converted to NADH (nicotinamide adenine dinucleotide) and FADH2 (flavin adenine dinucleotide 2) to generate ATP (adenosine triphosphate) in mitochondria [106]. Since FFAs are consumed during this process, the level of FFAs β-oxidation is closely related to lipid metabolism [106,107]. Luo et al., in 2019 reported that crocin enhanced the expression of genes involved in FFAs β-oxidation, including PPARα, acyl-CoA oxidase 1 (Acox1), carnitine palmitoyltransferase 1 (Cpt1), and 3-hydroxy-3-methylglutaryl-CoA synthase 2 (Hmgcs2) [75]. They found that oral administration of crocin for 8 weeks promoted FFAs β-oxidation via the AMPK (adenosine monophosphate (AMP)-activated protein kinase)-dependent pathway and improved fatty liver and metabolic profile in diabetic db/db mice [75]. Xie and colleagues also reported that oral administration of crocin-1 (a major member of the crocin family) enhanced hepatic fatty acid β-oxidation in mice [72]. Furthermore, a more recent animal study explored the possible role of crocin in lipid metabolism and obesity and found that crocin ameliorates obesity and increases lipid metabolism [108]. This study revealed that crocin increases fatty acid oxidation via the PPARα pathway and lowers ACC, FAS, and Srebp1c (sterol regulatory element-binding protein 1c) expression in HFD-induced obese mice [108].

Crocin has been shown to decrease lipogenesis in differentiated adipocytes [76]. Its protective effects against hyperlipidemia and obesity may be exerted via the AMPK signaling pathway, as the effects of crocin are suppressed when AMPK inhibitors are used [76]. AMPK is a key factor and central mediator of metabolism, and its disturbance is associated with obesity [75,76]. Fang et al., in 2020 proposed a connection between AMPK and PPAR-γ, suggesting that AMPK may decrease CDK5, resulting in reduced phosphorylation of PPAR-γ, which in turn decreases adipogenesis [76]. Gu et al., in 2018 reported that crocin reduces the gene expression of CCAAT/enhancer binding protein α (CEBPα), CEBPβ, PPAR-γ, aP2, FAS, and CD36, which are involved in adipogenesis. Additionally, it increases the gene expression of PPARα, lipoprotein lipase (LPL), and hormone-sensitive lipase (HSL), which are involved in lipolysis [73]. Inhibition of AMPK using BML-275 stops these effects of crocin. It can be concluded that the AMPK pathway is activated by crocin, inhibiting adipogenesis and elevating lipolysis. Furthermore, a CREB/BDNF signaling pathway may be involved in these therapeutic effects of crocin [109,110]. These pieces of evidence strongly suggest that crocin can modulate adipogenesis and adipolysis through several pathways, thereby preventing lipid accumulation and reducing dyslipidemia-related disorders (Fig. 3).

## 3 Lipid peroxidation and lipotoxicity

Lipid peroxidation, which refers to the oxidative degradation of lipids, is a pathological process where free radical species steal electrons from cellular membrane lipids. This process produces toxic byproducts such as malondialdehyde (MDA), F2-isoprostanes, 4-hydroxynonenal (4HNE), and thiobarbituric acid reactive substances (TBARS), which in turn induce oxidative damage to cellular elements such as lipids, proteins, and DNA [111]. These byproducts can bind with DNA, leading to mutagenic events by forming DNA adducts and induce the formation of toxic biomarkers like 8-oxo-2′-deoxyguanosine (8oxodG) [112,113]. Lipid peroxidation is a major marker of oxidative damage and is associated with various dyslipidemia-related disorders [111]. Therefore, preventing or reducing lipid peroxidation is of great importance to prevent subsequent cellular and tissue damage [112].

Crocin has been shown to inhibit free radical production and apoptosis, decrease lipid peroxidation, and increase glutathione levels and antioxidant enzyme activities [77]. Hepatic malondialdehyde (MDA) level is a marker of lipid peroxidation. Administration of crocin improved increased MDA levels and decreased superoxide dismutase (SOD) levels, which are observed in nonalcoholic steatohepatitis, indicating the antioxidant effects of crocin [114]. Lipid deposition in hepatocytes leading to hepatic steatosis is a common problem in nonalcoholic fatty liver disease, and steatotic hepatocytes are more susceptible to oxidative stress. Injection of crocin for 3 weeks in mice significantly decreased oxidative stress and Fas death receptor activities, improved lipid profiles, and normalized serum levels of hepatic enzymes [114]. Crocin also increases antioxidant activities and ameliorates disturbances caused by methotrexate in rats [115]. Moreover, it reduces the disorders caused by cisplatin-induced lipid peroxidation by decreasing MDA levels and increasing glutathione, glutathione peroxidase (GPx), catalase, and SOD levels [116]. In rats injected with cisplatin, crocin improved testicular lipid peroxidation and increased SOD levels [117]. Another chemotherapeutic drug, doxorubicin, enhances triglyceride (TG), low-density lipoprotein (LDL), very low-density lipoprotein (VLDL), MDA, and total oxidant status, while decreasing glutathione, SOD, catalase, and total antioxidant status. An animal study showed that co-injection of crocin with doxorubicin significantly improved the lipid profile and oxidative stress activities in rats [118].

In addition, crocin has demonstrated neuroprotective effects against certain neurotoxic agents [119]. Khalaf et al., in 2022 investigated the effects of co-administration of dapagliflozin, crocin, and some probiotics in STZ-induced diabetic rats. They observed significantly reduced oxidative, inflammatory, and apoptotic disorders, leading to an improved function of the gut microbiota, which in turn modulates the lipid profile [120]. Another study by Yousefsani et al., in 2021 reported a decrease in mitochondrial lipid peroxidation following crocin treatment in rats, indicating the protective effects of crocin against neurotoxicity [121]. d-galactose is commonly used as an animal model for aging and it increases the serum levels of alanine aminotransferase (ALT), aspartate aminotransferase (AST), alkaline phosphatase (ALP), MDA, and iNOS in rats. Crocin has been shown to ameliorate the levels of AST, ALT, ALP, MDA, and iNOS, suggesting its hepatoprotective properties against d-galactose-induced toxicity [122]. Similar hepatoprotective effects of crocin, attributed to the decrease in lipid peroxidation and potentiation of antioxidant enzymes, have been observed in other studies [78,79].

**Fig. 4:**
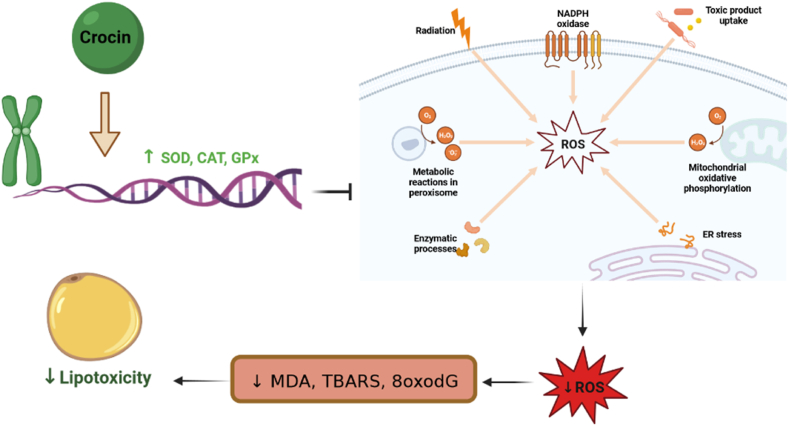
Crocin up-regulates SOD, CAT and GPx genes and potentiates antioxidant defense system which in turn decreases free radical generation and reduces toxic by-products production and so, ameliorates lipotoxicity (SOD = superoxide dismutase, CAT = catalase, GPx = glutathione peroxidase, TBARS = thiobarbituric acid reactive substances, MDA = malondialdehyde, 8oxodG = 8-oxo-2′-deoxyguanosine, ROS = reactive oxygen species).

The up-regulation of the Keap1-Nrf2/HO-1 signaling pathway has been proposed as a mechanism for the protective effects of crocin against the cardiotoxic side effects of arsenic trioxide [123]. Crocin may also exert protective effects against methylphenidate-induced neurotoxicity by strengthening the cyclic AMP response element-binding protein brain-derived neurotrophic factor (CREB/BDNF) system [109]. It has been shown to reverse the enhancing effects of methamphetamine on apoptosis, oxidative stress, and inflammation, while increasing P-CREB and BDNF activity [110]. The neuroprotective effect of crocin via the CREB/BDNF pathway has also been observed in methamphetamine-induced neurodegeneration in the hippocampus of rats [124]. Overall, crocin's potent antioxidant properties enable it to combat lipid peroxidation through multiple pathways and prevent subsequent injuries (Fig. 4).

## 4 Lipid absorption/transport and cholesterol metabolism

Plasma lipoproteins, including HDL, LDL, and VLDL, are involved in lipid transport, and an imbalance in their plasma levels may lead to dysregulated lipid metabolism and an increased risk of metabolic diseases [101]. A study conducted on a weight-loss herbal formula showed that crocin may act as a lipase activator, resulting in the suppression of lipid absorption and weight loss [125]. Additionally, Luo et al. reported that crocin decreased triglyceride (TG), total cholesterol (TC), and non-esterified fatty acids via an AMPK-dependent signaling pathway [74]. LDL is a primary carrier of cholesterol in plasma, and increased levels are positively associated with an enhanced risk of cardiovascular problems [101]. Wani et al. reported that crocin decreases the glycation of LDL, indicating a protective role of crocin against coronary artery diseases [126]. Moreover, crocin decreases the level of Ox-LDL and exhibits anti-atherosclerotic effects [127]. Sheng et al. reported that crocin treatment significantly reduced serum levels of TG, TC, LDL, and VLDL in diet-induced hyperlipidemic rats. They suggested that these lipid benefits of crocin may be exerted by inhibiting pancreatic lipase [128]. Crocin increases the serum level of HDL and decreases cholesterol, VLDL, LDL, and TG in rats receiving isoprenaline [129]. Furthermore, it reduces cholesterol and TG in the serum of mice with tumors, breast tumors, and breast cancer cell lines [130].

We have accumulated more evidence suggesting that crocin modulates the levels of lipid transporters. In mice, treatment with crocin for 56 days resulted in decreased serum triglyceride (TG) levels and circulating lipopolysaccharide [131]. Similarly, in STZ-induced diabetic rats, intraperitoneal injection of crocin for 25 days led to decreased blood sugar, cholesterol, TG, and LDL-C levels, while increasing body weight and HDL levels [132]. Zhang et al. reported that crocin reduced lipid deposition, upregulated HDL, and down-regulated total cholesterol (TC) and LDL levels [80]. Furthermore, they demonstrated that crocin inhibited foam cell formation and enhanced reverse cholesterol transport through the liver X receptor-α (LXR-α) and PPAR-γ pathways [80]. Xie et al., in 2019 conducted a study using corticosterone-treated animals and found that crocin significantly reduced the expression of LDL receptors in the liver, potentially resulting in lower levels of cholesterol elimination. Crocin reversed this effect by increasing the expression of fatty acid synthase and LDL receptors (LDL-R) [133]. Similar increasing effects of crocin on LDL-R and peroxisome proliferator-activated receptor-γ (PPAR-γ) gene expression have been observed in other studies [81]. PPAR-γ is a nuclear receptor and transcription factor involved in lipid and cholesterol metabolism. The overexpression of PPAR-γ following crocin treatment has been proposed as a possible mechanism for the therapeutic effects of crocin on lipid and cholesterol metabolism [129].

**Fig. 5:**
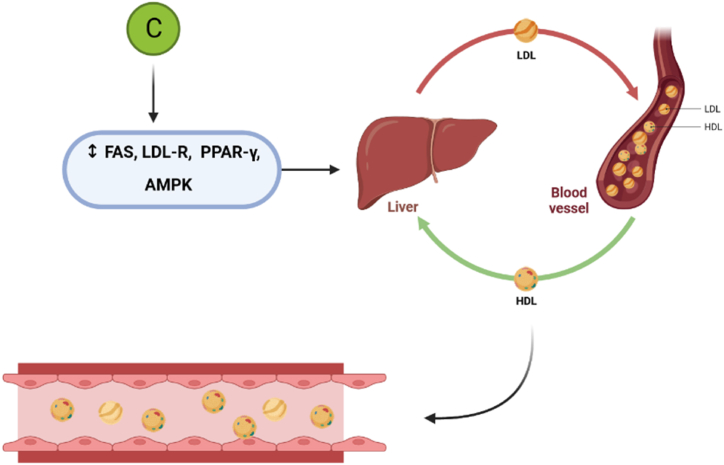
While LDL transports cholesterol from liver to the tissues, HDL transports excess cholesterol from tissues to liver and reduces its circulating level. Crocin induces these processes and help to normalizing plasma cholesterol level via different pathways (FAS = fatty acid synthase, LDL-R = LDL receptor, PPAR- γ = peroxisome proliferator activated receptor-γ, AMPK = adenosine mono phosphate activated protein kinase).

Additionally, there is clinical evidence supporting the lipid benefits of crocin on LDL and cholesterol homeostasis. A clinical study conducted on patients undergoing methadone maintenance treatment demonstrated that crocin administration for 8 weeks decreased fasting blood glucose (FBG), insulin levels, insulin resistance, TG, VLDL, total cholesterol, and malondialdehyde (MDA) levels, while increasing insulin sensitivity [81]. Similarly, an 8-week treatment with crocin increased serum HDL-C uptake capacity in patients with metabolic syndrome [134]. Another clinical trial showed that crocin increased HDL levels and decreased LDL, TG, and cholesterol levels, while also exerting protective effects against increased levels of IL-6 and TNF-α [135]. The results of a clinical trial indicated lower TG levels, but not TC, HDL, and LDL, following 90 days of treatment with a crocin supplement in patients with type 2 diabetes mellitus [136]. Collectively, these pieces of evidence strongly suggest that crocin has potent modulatory effects on cholesterol homeostasis and lipid absorption, potentially leading to an improved lipid profile (Fig. 5).

## 5 Conclusion

Dyslipidemia poses a significant challenge for individuals with and without diabetes, and it is a leading cause of cardiovascular complications in both populations. Therefore, the improvement of lipid metabolism and the maintenance of lipid homeostasis are of utmost importance to prevent these complications. Crocin, a naturally occurring pigment, has been recognized for its potent antioxidant and anti-inflammatory properties. However, emerging evidence suggests that crocin also offers metabolic benefits in lipid homeostasis, thereby potentially reducing lipid accumulation and mitigating cardiovascular issues. From screening and surveying the relevant studies, it is possible to aggregate the lipid-regulating mechanisms by crocin in three main points/domains: adipogenesis and adipolysis, lipid peroxidation, and lipid and cholesterol transport and metabolism. The main limitation of the current extant evidence is lack of clinical findings from well-designed randomized controlled trials. Given the encouraging results from experimental studies, clinical trials using crocin in diabetic and non-diabetic individuals with dyslipidemia are warranted to advance our knowledge. However, crocin may serve as an effective nutraceutical for normalizing lipid metabolism, reducing lipid accumulation, and combating obesity. Ultimately, these effects may significantly alleviate dyslipidemia-related disorders.

## Funding

None.

## Data availability

There is no primary data associated with this review article.

## CRediT authorship contribution statement

**Habib Yaribeygi:** Writing – original draft, Conceptualization. **Mina Maleki:** Writing – review & editing. **Farin Rashid-Farrokhi:** Writing – review & editing. **Payman Raise Abdullahi:** Writing – review & editing. **Mohammad Amin Hemmati:** Writing – review & editing. **Tannaz Jamialahmadi:** Writing – review & editing. **Amirhossein Sahebkar:** Writing – review & editing, Conceptualization.

## Declaration of competing interest

The authors declare that they have no known competing financial interests or personal relationships that could have appeared to influence the work reported in this paper.
